# Hyperoxemia in postsurgical sepsis/septic shock patients is associated with reduced mortality

**DOI:** 10.1186/s13054-021-03875-0

**Published:** 2022-01-10

**Authors:** Marta Martín-Fernández, María Heredia-Rodríguez, Irene González-Jiménez, Mario Lorenzo-López, Estefanía Gómez-Pesquera, Rodrigo Poves-Álvarez, F. Javier Álvarez, Pablo Jorge-Monjas, Juan Beltrán-DeHeredia, Eduardo Gutiérrez-Abejón, Francisco Herrera-Gómez, Gabriella Guzzo, Esther Gómez-Sánchez, Álvaro Tamayo-Velasco, Rocío Aller, Paolo Pelosi, Jesús Villar, Eduardo Tamayo

**Affiliations:** 1 Department of Medicine, Toxicology and Dermatology, University of Valladolid, Valladolid, Spain; 2 BioCritic, Group for Biomedical Research in Critical Care Medicine, Valladolid, Spain; 3 Centro de Investigación Biomédica en Red de Enfermedades Infecciosas (CIBERINFEC), Instituto de Salud Carlos III, Madrid, Spain; 4 Department of Anaesthesiology and Critical Care, Hospital Clínico Universitario de Salamanca, Salamanca, Spain; 5 Department of Anaesthesiology and Critical Care, Hospital Clínico Universitario de Valladolid, Valladolid, Spain; 6 Department of Pharmacology, University of Valladolid, Valladolid, Spain; 7 Department of Surgery, University of Valladolid, Valladolid, Spain; 8 Transplantation Center, Lausanne University Hospital and University of Lausanne, Lausanne, Switzerland; 9 Department of Hematology, Hospital Clínico Universitario de Valladolid, Valladolid, Spain; 10 Department of Gastroenterology, Hospital Clínico Universitario de Valladolid, Valladolid, Spain; 11 Department of Surgical Sciences and Integrated Diagnostics, University of Genoa, Genoa, Italy; 12 IRCCS for Oncology and Neurosciences, San Martino Policlinico Hospital, Genoa, Italy; 13 CIBER de Enfermedades Respiratorias, Instituto de Salud Carlos III, Madrid, Spain; 14 Research Unit, Hospital Universitario Dr. Negrín, Barranco de la Ballena s/n, 4th Floor-South Wing, 35019 Las Palmas de Gran Canaria, Spain; 15 Li Ka Shing Knowledge Institute at St. Michael’s Hospital, Toronto, ON Canada

**Keywords:** Hyperoxemia, Outcome, Sepsis, Septic Shock, Surgical patients, Infection

## Abstract

**Background:**

Despite growing interest in treatment strategies that limit oxygen exposure in ICU patients, no studies have compared conservative oxygen with standard oxygen in postsurgical patients with sepsis/septic shock, although there are indications that it may improve outcomes. It has been proven that high partial pressure of oxygen in arterial blood (PaO_2_) reduces the rate of surgical-wound infections and mortality in patients under major surgery. The aim of this study is to examine whether PaO_2_ is associated with risk of death in adult patients with sepsis/septic shock after major surgery.

**Methods:**

We performed a secondary analysis of a prospective observational study in 454 patients who underwent major surgery admitted into a single ICU. Patients were stratified in two groups whether they had hyperoxemia, defined as PaO_2_ > 100 mmHg (*n* = 216), or PaO_2_ ≤ 100 mmHg (*n* = 238) at the day of sepsis/septic shock onset according to SEPSIS-3 criteria maintained during 48 h. Primary end-point was 90-day mortality after diagnosis of sepsis. Secondary endpoints were ICU length of stay and time to extubation.

**Results:**

In patients with PaO_2_ ≤ 100 mmHg, we found prolonged mechanical ventilation (2 [8] vs. 1 [4] days, *p* < 0.001), higher ICU stay (8 [13] vs. 5 [9] days, *p* < 0.001), higher organ dysfunction as assessed by SOFA score (9 [3] vs. 7 [5], *p* < 0.001), higher prevalence of septic shock (200/238, 84.0% vs 145/216) 67.1%, *p* < 0.001), and higher 90-day mortality (37.0% [88] vs. 25.5% [55], *p* = 0.008). Hyperoxemia was associated with higher probability of 90-day survival in a multivariate analysis (OR 0.61, 95%CI: 0.39–0.95, *p* = 0.029), independent of age, chronic renal failure, procalcitonin levels, and APACHE II score > 19. These findings were confirmed when patients with severe hypoxemia at the time of study inclusion were excluded.

**Conclusions:**

Oxygenation with a PaO_2_ above 100 mmHg was independently associated with lower 90-day mortality, shorter ICU stay and intubation time in critically ill postsurgical sepsis/septic shock patients. Our findings open a new venue for designing clinical trials to evaluate the boundaries of PaO_2_ in postsurgical patients with severe infections.

**Supplementary Information:**

The online version contains supplementary material available at 10.1186/s13054-021-03875-0.

## Background

Sepsis and septic shock are major causes of mortality in surgical patients [1]. It is estimated that up to one-third of total cases of sepsis are among surgical patients [2]. More than 1.7 million people in the United States (US) are diagnosed with sepsis each year [3], which causes 270,000 deaths [4] and accounts for the highest hospital cost [5]. In Spain, sepsis incidence and associated mortality increased from 200 cases and 56 deaths per 100,000 inhabitants in the year 2000 to 480 cases and 830 deaths per 100,000 population in 2013, respectively [6].

Septic shock patients admitted to the ICU consume many healthcare resources and require early management and treatment [7]. In the pathophysiology of shock, there is an imbalance between oxygen supply and oxygen consumption [8]. Therefore, one of the mainstays of treatment for septic shock is oxygen administration. However, the appropriate regimen of oxygen administration is unknown and the Surviving Sepsis Guidelines do not provide a formal recommendation for oxygen targets [7]. On the other hand, the effects of hyperoxaemia may also be beneficial by enhancing host defense against extracellular pathogens by neutrophils [9]. Neutrophils’ bactericidal activity is mediated by oxidative killing, a crucial defense mechanism against surgical pathogens [10]. This potent bactericidal mechanism is dependent on the production of superoxide radicals from molecular oxygen [11].

Several studies have shown that the administration of oxygen for achieving high partial pressure of oxygen in arterial blood (PaO_2_) reduces the rate of surgical-wound infections and mortality in patients under major surgery [11–13]. A recent meta-analysis of 17 randomized controlled trials involving more than 8000 patients concluded that perioperative hyperoxia (FiO_2_ 0.80) reduced the risk of surgical site infections in colorectal surgery [14]. Few studies have addressed the assessment of hyperoxaemia in sepsis and their results do not shed light on the problem [14–17]. A post-hoc analysis of the HYPERS2S study found that hyperoxaemia may be associated with increased 90-day mortality [16] and, in contrast, a post-hoc analysis of the ICU-ROX study found that conservative oxygen therapy increases 90-day mortality in patients with sepsis [17].

In view of the paucity of clinical evidence, the debate on the optimal oxygen concentration in sepsis/septic shock patients has only just begun. In this study we performed an exploratory post hoc analysis to evaluate the effect on 90-day mortality of conservative versus standard oxygen therapy administered during the first 48 h after the onset of sepsis/septic shock. This information may help to both guide the performance of a future clinical trial and test the hypothesis that hyperoxemia would improve outcome compared to conservative oxygen therapy in patients with sepsis/septic shock.

## Methods

### Patient population

This is a secondary analysis of a prospective cohort of 454 adult patients (≥ 18 years old) who underwent major surgery and were admitted to the Surgical ICU of the 700-bed Hospital Clínico Universitario, Valladolid, Spain, from December 2006 to February 2017, with a diagnosis of sepsis or septic shock according to SEPSIS-3 definitions and need for invasive mechanical ventilation [18]. Patients meeting clinical criteria for sepsis/septic shock with a negative microbiological culture were excluded. The study protocol was approved by the Ethics Committee for Clinical Research, Hospital Clínico Universitario de Valladolid, Valladolid, Spain (approval No. PI 20-2070). This study followed current Spanish legislation for biomedical research, fulfilling the standards indicated by the Declaration of Helsinki. Written informed consent was obtained from patients, patients' relatives or their legal representative before enrolment.

We stratified patients into two groups according to the value of PaO_2_ on the day of sepsis or septic shock onset, maintained during 48 h: (i) hyperoxemia (PaO_2_ > 100 mmHg) group, or (ii) normoxemia (PaO_2_ ≤ 100 mmHg) group [19].

Patients were managed and treated (Additional file 1: File 1) according to current guidelines for general critical care management [18], which include the following: (i) early identification of causative microorganism, optimization of intravenous antibiotic selection and timely administration on the basis of antibiogram; (ii) fluid resuscitation and vasopressor use were individualized with the goal of maintaining a systolic blood pressure ≥ 90 mmHg or a mean arterial pressure ≥ 65 mmHg; (iii) to maintain hemoglobin between 7 and 10 g/dL according to the patient’s overall clinical status [7].

The choice of drugs for sedation and analgesia, hemodynamic management modalities, and the decision to perform tracheostomy were left to the discretion of the attending physician. Weaning from the ventilator was started when the attending physician considered it clinically appropriate. Gastric protection was routinely performed with omeprazole (20 mg/iv) during the first 24 h of ICU stay.

### Data collection and follow-up

Patients admitted into the ICU were screened daily during the study period to identify eligible patients and determine the onset of severe sepsis/septic shock. We used a specific standardized form to collect demographic and clinical data, including hematological, biochemical, radiological, microbiological, and biomarker levels in the first 24 h after diagnosis of sepsis/septic shock. Severity of illness was assessed using the Sequential Organ Failure Assessment (SOFA) [20] and the Acute Physiology and Chronic Health Evaluation II (APACHE II) [21] scores. Sepsis and septic shock were diagnosed according to the 3rd International Consensus Definitions for Sepsis and Septic Shock (Sepsis-3) criteria [18]. Sepsis was defined as life-threatening organ dysfunction caused by a dysregulated host response to infection. Organ dysfunction can be represented by an increase in the SOFA score of ≥ 2 points. Septic shock is a subset of sepsis that can be identified by a vasopressor requirement to maintain a mean arterial pressure ≥ 65 mmHg and serum lactate > 2 mmol/L (> 18 mg/dL) in the absence of hypovolemia. We followed the criteria of the Centers for Disease Control and Prevention (CDC) [22] for the diagnosis of nosocomial infections during ICU stay (Additional file 1). We checked that no patient had any infection before the major surgical intervention and that all septic patients had a confirmed source of infection.

### Clinical end-points and statistical analysis

The primary endpoint in both groups was mortality at 90 days after diagnosis of sepsis/septic shock. Secondary endpoints were length of ICU stay and time to extubation.

Sample size calculation was based on the percentage of surgical patients with a PaO_2_ above or below 100 mmHg following a meaningful methodological perspective. Assuming a 90-day mortality of 25% and 40% respectively, a risk alpha of 5%, a power of 80% in a bilateral contrast and estimating a loss rate of 30%, a sample size of 216 subjects in each group was estimated. Differences between groups were assessed using $\chi^{2}$ test for categorical variables and the Mann Whitney U test for continuous variables. We analyzed probability of death to day-90 after sepsis diagnosis in both groups using Kaplan–Meier curves and tested with the log-rank test (Mantel–Haenszel). The relationship between PaO_2_ and mortality was plotted to examine the dose–response curve [23]. We considered 2-sided *p-*values < 0.05 to indicate statistical significance. The potential association between PaO_2_ levels and risk of 90-day mortality was further evaluated by using a multivariate logistic regression analysis. Potential confounding factors for logistic regression were identified from variables described in Table 1. Those variables yielding a *p*-value < 0.1 in the univariate regression analysis were included in the multivariate analysis as adjusting variables. A sub-analysis following this methodology was performed excluding patients with severe hypoxemia (PaO_2_ < 60 mmHg) at the time of inclusion into the study. We calculated the optimal operating point (OOP) of the multivariate regression model, being the value for which the point on the curve had the minimum distance to the upper left corner (where sensitivity = 1, and specificity = 1). By Pythagoras’ theorem this distance is: Optimal Operating Point (OOP) $= \surd \left( {1 - {\text{sensitivity}}} \right)^{2} + \left( {1 - {\text{specificity}}} \right)^{2}$. All data were analyzed using the IBM SPSS 22.0 software (SPSS, Chicago, IL) and R version 3.0.1 (R Foundation for Statistical Computing, Vienna, Austria).

**Table 1:** Baseline characteristics of patients

	PaO_2_ ≤ 100 mmHg (1) (*n* = 238)	PaO_2_ > 100 mmHg (2) (*n* = 216)	*p* value (1 vs. 2)
Characteristics			
Age [years, median [IQR]]	73 [14]	72 [16]	0.69
Male [%, (n)]	65.5 (156)	54.6 (118)	0.028
Comorbidities [% (n)]			
Chronic cardiovascular disease	34.9 (83)	27.8 (60)	0.09
Chronic respiratory disease	22.3 (53)	12.5 (27)	0.005
High blood pressure	59.7 (142)	54.6 (118)	0.22
Chronic renal failure	8.4 (20)	9.7 (21)	0.66
Chronic hepatic failure	3.3 (8)	4.2 (9)	0.67
Diabetes mellitus	22.7 (54)	22.2 (48)	0.86
Cancer	27.3 (65)	39.4 (85)	0.008
Immunosuppression	4.6 (11)	3.7 (8)	0.61
Obesity	18.5 (44)	11.1(24)	0.024
Surgery type, [% (n)]			
Abdominal	60.9 (145)	76.4 (165)	0.009
Cardio-thoracic	2.9 (7)	3.2 (7)	0.98
Vascular	6.3 (15)	3.2 (7)	0.07
Urological/renal	2.1 (5)	2.3 (5)	0.99
Other	4.6 (11)	2.3 (5)	0.12
Source of infection, [% (n)]			
Respiratory tract	32.9 (73)	16.3 (33)	< 0.001
Abdomen	50.0 (111)	61.9 (125)	0.014
Urinary tract	3.4 (8)	4.6 (10)	0.50
Surgical site	0.8 (2)	1.4 (3)	0.58
Bacteremia	3.8 (9)	3.2 (7)	0.74
Other	11.3 (27)	11.6 (25)	0.96
Microbiology, [% (n)]			
Gram +	29.4 (70)	29.2 (63)	0.95
Gram −	38.2 (91)	31.0 (67)	0.11
Fungi	16.4 (39)	10.6 (23)	0.08
Measurements at diagnosis [median [IQR]]			
PaO_2_ (mmHg)	74.15 [25]	134 [45]	< 0.001
FiO_2_	0.50 [0.05]	0.50 [0]	0.47
PaO_2_/FiO_2_ ratio (mmHg)	157.64 [73.45]	278 [108.50]	< 0.001
PaO_2_ at 48 h (mmHg)	71.50 [25]	131 [45]	< 0.001
Total bilirubin (mg/dl)	0.89 [1.06]	0.75 [0.82]	0.12
Glucose (mg/dl)	168 [76.15]	171 [75.25]	0.82
Creatinine (mg/dl)	1.90 [2.51]	1.51 [1.73]	0.001
Na (mmol/L)	137 [6]	136 [5]	0.024
K (mmol/L)	4 [1.1]	4 [1]	0.54
Platelet count (cells/mm^3^)	173,000 [155500]	197,000 [184000]	0.014
Lactate (mmol/L)	2.66 [2.17]	2.20 [2.20]	0.08
Procalcitonin (ng/ml)	5.50 [16.28]	5.05 [23.41]	0.61
C-Reactive Protein (mg/L)	250.55 [176.50]	232 [155.40]	0.039
White Blood cells (cells/mm^3^)	13,660 [12295]	12,955 [10521]	0.06
Lymphocytes (cells/mm^3^)	961.65 [988.14]	767.70 [901.52]	0.003
Neutrophils (cells/mm^3^)	11,727.31 [11263.94]	11,308.80 [9629.29]	0.16
Urgent surgery	66.4 (158)	80.1 (173)	0.001
SOFA score	9 [3]	7 [5]	< 0.001
APACHE II score	16 [7]	14 [7]	< 0.001
Time course and outcome			
Length of hospital stay [days, median (IQR)]	25 [24]	23.50 [27]	0.97
Length of ICU stay [days, median (IQR)]	8 [13]	5 [9]	< 0.001
Length of mechanical ventilation [days, median (IQR)]	2 [8]	1 [4]	< 0.001
Sepsis [% (n)]	16.0 (38)	32.9 (71)	< 0.001
Septic shock [% (n)]	84.0 (200)	67.1 (145)	< 0.001
Mortality at 90 days [% (n)]	37.0 (88)	25.5 (55)	0.008

Continuous variables are represented as median and interquartile range (IQR); categorical variables are represented as percentages (%) and number (n). ICU: intensive care unit; SOFA: Sequential Organ Failure Assessment Score

## Results

A total of 238 patients had a PaO_2_ ≤ 100 mmHg and 216 patients had a PaO_2_ > 100 mmHg at study inclusion. Baseline characteristics of patients are reported in Table 1. We found that patients in the group of PaO_2_ ≤ 100 mmHg had higher organ dysfunction (SOFA score 9 [3] vs. 7 [5], *p* < 0.001), and prevalence of septic shock (84.0% [200] vs. 67.1% [145], *p* < 0.001. Also, higher levels of C-reactive protein (250.55 [176.50] vs. 232 [155.40], *p* = 0.039) (mg/L), and APACHE II score (16 [7] vs. 14 [7], *p* < 0.001) were more common in the group of patients with PaO_2_ ≤ 100 mmHg.

**Fig. 1:**
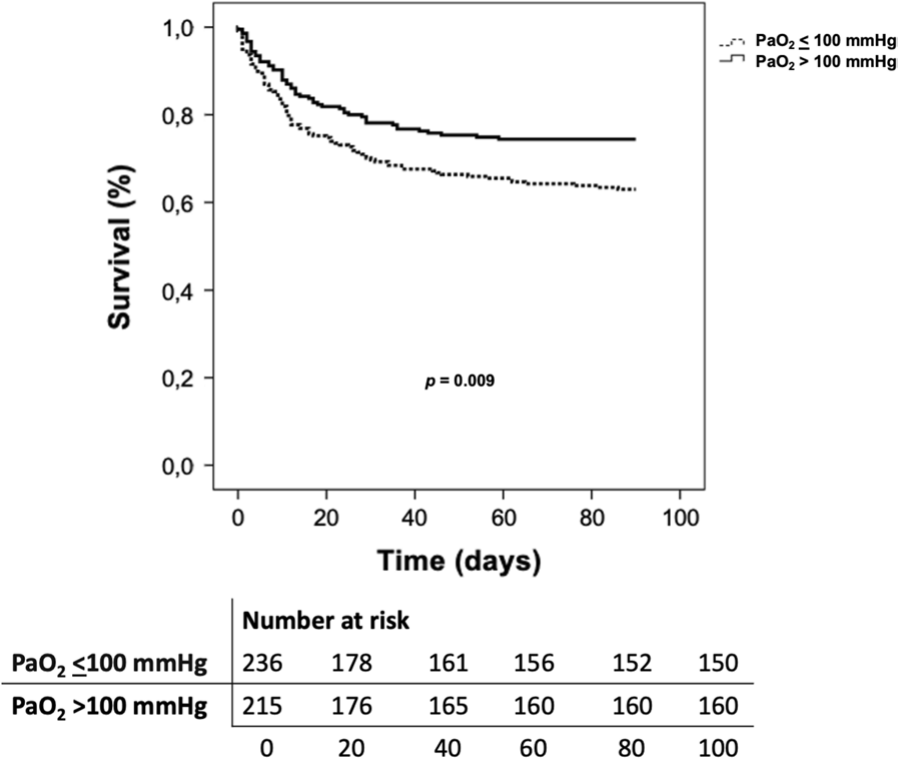
Kaplan–Meier survival curves for 90-day mortality

**Fig. 2:**
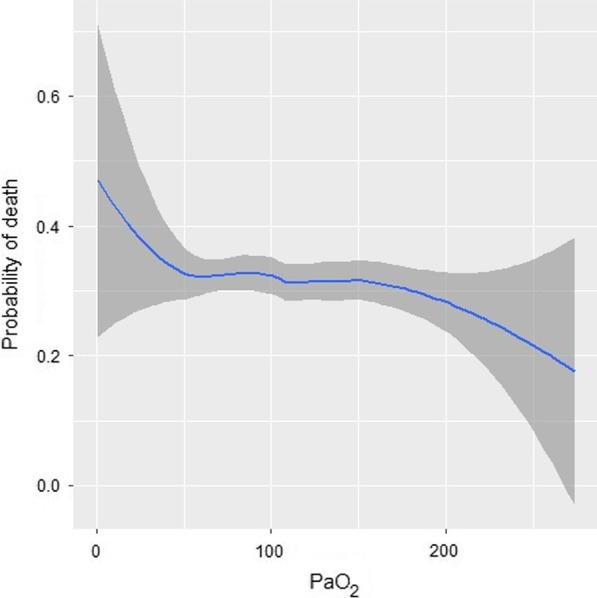
Adjusted probability of mortality by arterial oxygen levels. Grey zones represent 95% confidence intervals

Patients with PaO_2_ > 100 mmHg had lower 90-day mortality (25.5% [55] vs. 37.0% [88], *p* = 0.008), reduced length of ICU stay (5 [9] vs. 8 [13] days, *p* < 0.001) and shorter duration of mechanical ventilation (1 [4] vs. 2 [8] days, *p* < 0.001). On average, patients with PaO_2_ > 100 mmHg died 7.8 days later (mean survival time 63.3 vs 71.1 days) when assessing 90-day mortality (*p* = 0.009) (Fig. 1). A PaO_2_ > 100 mmHg was associated with higher probability of 90-day survival (OR 0.61, 95% CI 0.39–0.95, *p* = 0.029). This association was independent of age, presence of chronic renal failure, procalcitonin levels, or APACHE II score above 19 in the multivariate analysis (Table 2). The lower the value of PaO_2_, the greater the risk of death at 90 days (Fig. 2).

**Table 2:** Univariate and multivariate analysis for evaluating the risk of mortality at 90 days

	Univariate analysis				Multivariate analysis			
	OR	[CI 95%]		*p*	OR	[CI 95%]		*p*
Age	1.04	1.02	1.06	< 0.001	1.03	1.01	1.05	0.008
Chronic renal failure	2.84	1.48	5.44	0.002	2.93	1.44	5.96	0.003
PCT (ng/mL) Ln	1.22	1.07	1.37	0.002	1.16	1.02	1.33	0.029
APACHE II > 19	3.74	2.26	6.21	< 0.001	2.96	1.72	5.11	< 0.001
PaO_2_ > 100 mmHg	0.58	0.39	0.87	0.009	0.61	0.39	0.95	0.029

CI, Confidence interval; OR, odds ratio; PCT, procalcitonin; APACHE II, acute physiology and chronic health evaluation II

On average, patients with PaO_2_ > 100 mmHg had lower ICU stay (mean values 7.7 vs 10.6 days) when assessing 28-day ICU stay (*p* = 0.001) (Additional file 1: Fig. S1). Patients with PaO_2_ > 100 mmHg were extubated 1.7 days earlier (mean values 4.0 vs 5.7) when assessing a 28-day intubation period (*p* = 0.022) (Additional file 1: Fig. S1). The optimal operating point (OOP) of the multivariate analysis has a sensitivity of 63.4% and a specificity of 68.9% for predicting mortality. Patients with levels over this OOP died 22 days before on average (mean survival time 75.9 vs 54.2 days) (Additional file 1: Fig. S2). These results were confirmed when a further sub-analysis was performed excluding more severely hypoxemic patients (PaO_2_ < 60 mmHg) at the time of study inclusion (Additional file 1: Table S1, Figs. S3 and S4).

## Discussion

In the present study in 454 patients with sepsis/septic shock, a PaO_2_ > 100 mmHg during the first 48 h after major surgery was associated with a lower risk of 90-day mortality, lower length of ICU stay and reduced time to extubation. Similar results were obtained when patients with PaO_2_ < 60 mmHg were excluded. Our findings could have an important clinical relevance for reducing overall mortality in postoperative patients developing sepsis/septic shock.

Hyperoxemia and hypoxemia have been associated with increased mortality in mechanically ventilated patients [24–27]. A recent meta-analysis in critically ill patients suggested a reduction in overall mortality with the use of conservative [median baseline SpO_2_ 96.7% (range 93.4–98.0%; IQR 95.0–97.0)] compared to liberal oxygen therapy [median baseline SpO_2_ 96.4% (range 94.0–99.0%; IQR 95.8–97.8)] [28]. However, in different subpopulations of critically ill patients the evidence is less certain, and the optimal oxygen strategy is still unknown. This is also due to the fact that oxygen thresholds are different and arbitrary among studies [29]. In patients with cardiac ischaemia or stroke, recent guidelines stated a strong recommendation against the use of oxygen therapy in non-hypoxaemic patients [peripheral oxygen saturation (SpO_2_) ≥ 93%] [30]. An association between severe hyperoxemia (PaO_2_ > 300 mmHg) [31] and increased mortality has been reported in patients where lung function was relatively preserved compared to either hypoxemia (PaO_2_ < 60 mmHg) or normoxemia (PaO_2_ 60–300 mmHg), such as after cardiac arrest [31–34], stroke [35], and traumatic brain injury [36–38]. Helmerhorst et al. [39] found that hyperoxia [the most commonly used threshold was 300 mmHg (range, 85–487 mm Hg)] was associated with poor hospital outcome in a meta-analysis, including various subsets of critically ill patients from heterogeneous studies. On the other hand, Vaahersalo et al. [40] described an improved long-term outcome with moderate hyperoxia (PaO_2_ 128–237 mmHg) in cardiac arrest, while other authors did not show any difference in several biomarkers of brain injury comparing a PaO_2_ of 75–113 mmHg to 150–188 mmHg [41, 42].

The association between hyperoxemia and outcome in sepsis/septic shock also remains to be unclear [43–46]. A post-hoc analysis of the HYPERS2S study revealed that hyperoxia (FiO_2_ 1.0) in comparison to normoxia (FiO_2_ set to target an arterial oxygen saturation of 88–95%) may be associated with increased 90-day mortality [16]. However, a recent study evaluating the effects of hyperoxemia (PaO_2_ > 120 mmHg) vs. normoxemia (PaO_2_ between 70–120 mmHg) on mortality in mechanically ventilated ICU patients with septic shock found no impact on mortality in this patient population [15]. In the ICU-ROX trial [45], conservative oxygen therapy [oxygen saturation measured by pulse oximetry (SpO_2_) between 90 and 97%] did not show differences when compared to liberal oxygen therapy (there was no protocol-defined upper limit of SpO_2_) in terms of ventilator-free days and mortality at 90 and 180 days. These results are in contrast with those from a previous trial in which conservative oxygen therapy (PaO_2_ 70–100 mmHg) was associated with a lower rate of deaths in comparison with conventional control group (PaO_2_ up to 150 mmHg) [44]. On the other hand, a post-hoc analysis of the ICU-ROX study revealed that higher PaO_2_ treatment in sepsis/septic shock patients was associated with a reduction in 90-day mortality, in line with our findings, while conservative oxygen therapy may be harmful [17]. In addition, the LOCO-2 study conducted in ARDS patients did not report differences in mortality at 28 days when using a conservative oxygenation therapy (PaO_2_ 55–70 mmHg; SpO_2_ of 88 to 92%) vs. liberal oxygen therapy (PaO_2_ 90–105 mmHg; SpO_2_ ≥ 96%) [47]. Gelissen et al. [48] reported that among critically ill patients with 2 or more SIRS criteria, treatment with a low-normal PaO_2_ target (PaO_2_ range of 60–90 mmHg) compared with a high-normal PaO_2_ target (PaO_2_ range of 105–135 mmHg) did not result in a reduction in organ dysfunction. Zhang et al. [49] reported that the effect of PaO_2_ on mortality risk is a quadratic function in sepsis, in which both low and high PaO_2_ were associated with a high mortality risk. Specifically, an increment in PaO_2_ up to 300 mmHg was associated with reduced risk of death, in agreement with our findings.

We also found a lower length of ICU stay and time to extubation in sepsis/septic shock patients with PaO_2_ above 100 mmHg. In contrast with our findings, Yamamoto et al. [50] found an association between hyperoxemia and longer ICU stay and time to extubation. However, they included a subset of trauma patients where hyperoxemia was defined as PaO_2_ ≥ 300 mmHg. Six et al. [51] found no association between hyperoxemia (SpO_2_ ≥ 98%) and ICU stay or time to extubation in critically ill patients with ventilator-associated pneumonia, but the definition used for hyperoxemia was based on an arbitrary threshold and the sample size was underpowered. In line with our findings, several studies have reported the potential role of supplemental perioperative oxygen to reduce the rate of surgical-wound infections in postsurgical patients [11–13].

The results of most studies are contradictory as they include heterogeneous patients using different oxygen thresholds. Most of these studies included patients with sepsis criteria but failed to isolate any germ in the cultures. Also, in those studies, the authors did not report whether the antibiotic was administered according to the antibiogram, nor reported the duration of antimicrobial stewardship. An important part of antimicrobial stewardship is to restrict antimicrobial therapy to the shortest course associated with best outcomes [52–56]. The optimal duration of antimicrobial therapy for a given sepsis/septic shock patient depends on several factors [57, 58]. Sepsis guidelines recommend a duration of antibiotic treatment to 3 days. It is understood that for an effective combination of oxygen, neutrophil function, and antibiotic it should last at least 48–72 h. Neutrophil dysfunction and low levels of partial pressure of tissue oxygen (PsqO_2_) could explain the failure of appropriate antibiotic treatment. Neutrophils are the final effectors cells of innate immunity with a primary role in the clearance of extracellular pathogens mediated by oxidative killing [9, 10]. Killing by oxidation is the most important defense mechanism against surgical pathogens, and depends on PsqO_2_ in contaminated tissue [11]. High PaO_2_ acts at systemic levels to increase partial pressure of tissue oxygen (PsqO_2_) [59]. The risk of infection is, therefore, inversely related to PsqO_2_ [59]. Furthermore, the presence of high PaO_2_ enhances neutrophil superoxide production, thus potentially improving pathogen killing by the innate immune system (Additional file 1: Fig. S5) [17]. During sepsis, oxygen delivery to the tissues may be impaired in some patients, making liberal provision of oxygen of great value. Thus, the use of high PaO_2_ might directly reduce the consequences of infections via enhanced oxidative killing of bacteria and indirectly in patients presenting wounds by re-epithelialization, blood vessel angiogenesis, and tissue collagen synthesis, which improves wound healing [17].

We acknowledge some limitations of our study. First, this is a secondary analysis of a prospective study to identify sepsis/septic shock patients and not for testing whether a threshold for PaO_2_ had any impact on hospital mortality in a randomized controlled study design. As a result, pathogen clearance and infection resolution were not evaluated because our study database was not designed to assess the effects of oxygen. Second, our study only monitored oxygen values for 48 h. Third, our study was conducted in a single center and should be tested in a multicenter fashion design to validate the potential role of PaO_2_ in predicting mortality in surgical patients with infection.

## Conclusions

In summary, oxygenation with a PaO_2_ above 100 mmHg was independently associated with lower 90-day mortality, shorter ICU stay and intubation time in critically ill postsurgical sepsis/septic shock patients. Our findings open a new venue for designing clinical trials to evaluate the boundaries of PaO_2_ in postsurgical patients with severe infections.

## Supplementary Information

**Additional file 1**: **File 1**. Supplementary methods regarding treatment, diagnosis and definitions of patients recruited for the study. **Figure S1.** (A) Kaplan–Meier curve for 28-day intubation. (B) Kaplan–Meier curve for 28-day ICU stay. **Figure S2.** Kaplan–Meier survival curves for 90-day mortality of the multivariate regression model. **Table S1.** Univariate and multivariate analysis for evaluating the risk of mortality at 90 days excluding patients with severe hypoxemia (PaO_2_ < 60 mmHg) at the time of inclusion into the study. **Figure S3.** Kaplan–Meier survival curves for 90-day mortality excluding patients with severe hypoxemia (PaO_2_ < 60 mmHg) at the time of inclusion into the study. **Figure S4. (**A) Kaplan–Meier curve for 28-day intubation excluding patients with severe hypoxemia (PaO_2_ < 60 mmHg) at the time of inclusion into the study. (B) Kaplan–Meier curve for 28-day ICU stay excluding patients with severe hypoxemia (PaO_2_ < 60 mmHg) at the time of inclusion into the study. **Figure S5.** Neutrophils’ bactericidal activity mediated by oxidative killing.

## Data Availability

The datasets used and/or analysed during the current study are available from the corresponding author on reasonable request.
