# Supplementary material for: Hyperoxemia in postsurgical sepsis/septic shock patients is associated with reduced mortality
Source: Crit Care. 2022 Jan 10;26:4. doi: 10.1186/s13054-021-03875-0 (PMC8744280; doi:10.1186/s13054-021-03875-0)

***Supplementary file***

**Hyperoxemia in postsurgical sepsis/septic shock patients is associated with reduced mortality**

* Marta Martín-Fernández, * María Heredia-Rodríguez, Irene González-Jiménez,

Mario Lorenzo-López, Estefanía Gómez-Pesquera, Rodrigo Poves-Álvarez,

F Javier Álvarez, Pablo Jorge-Monjas, Juan Beltrán-De Heredia, Eduardo Gutiérrez-Abejón, Francisco Herrera-Gómez, Gabriella Guzzo, Esther Gómez-Sánchez, Álvaro Tamayo-Velasco, Rocío Aller, ¶ Paolo Pelosi, ¶ Jesús Villar, ¶ Eduardo Tamayo

(*) These authors have equal contribution as first authors.

(¶) These authors have equal contribution as senior authors.

**Supp File 1. Supplementary methods regarding treatment, diagnosis and definitions of patients recruited for the study.**

**SUPPLEMENTARY METHODS**

**Treatment**

Patients were extubated when they were hemodynamically stable, had a Ramsay score of 2 to 3, a Tobin index (respiratory rate [spontaneous]/tidal volume [liters]) less than 105, a partial pressure of arterial oxygen (PaO_2_) >60 mmHg on a fraction of inspired oxygen (FiO_2_) <0.4, PaO_2_/FiO_2_ ratio >200 mmHg, a continuous positive airway pressure less than 5,1 cmH_2_O, a PaCO_2_ <50 mmHg with an arterial pH >7.35, and there was no significant bleeding. The administration of oxygen concentration was performed according to the criteria of the physician responsible for the patient.

Patients were managed and treated according to the standards for sepsis/septic shock following international guidelines [1], and based on our prior experience in identifying the most common bacterial pathogens associated with sepsis in our ICU. Appropriate empirical antibiotic treatment was defined when at least one of the drugs administered immediately after the performance of the microbiological diagnostic test was confirmed as effective against the pathogens obtained on the antibiogram. The only exception was in case of *Pseudomonas aeruginosa*, in which two effective drugs were required. Antibiotic administration included initial empirical treatment of methicillin-resistant *Staphylococcus aureus* with linezolid or teicoplanin and treatment of *Pseudomonas aeruginosa* with at least one of the following antibiotics: imipenem, cefepime, or piperacillin-tazobactam, in association with amikacin or ciprofloxacin. Mouthwashes with chlorhexidine were prescribed twice a day.

**Diagnostics and definitions**

Patients ventilated for >48 h were diagnosed with ventilator-associated pneumonia (VAP) if they had new and/or progressive pulmonary infiltrates on a chest radiograph, and two or more of the following criteria: fever (≥38.5ºC) or hypothermia (<36ºC), leukocytosis (≥12×10^9^/L), purulent tracheobronchial secretions, or a decrease in the PaO_2_/FiO_2_ ratio of at least 15% in the previous 48 h. Patients showing a Clinical Pulmonary Infection Score (CPIS) >6 were also included. Confirmation of diagnosis included the isolation of at least one pathogenic microorganism with a significant bacterial count (i.e. ≥103 colony-forming units (CFU)/mL obtained by telescopic brushing or endotracheal aspiration, respectively) [2].

Intra-abdominal infection was diagnosed based on: i) a positive culture from an abscess or purulent material from intraabdominal space, or ii) an abscess or other intraabdominal infection on gross anatomic or histopathologic exam, or iii) the presence of an abscess or other evidence of intraabdominal infection on gross anatomic or histopathologic exam and a positive culture, or iv) the presence of fever (>38.0°C), hypotension, nausea, vomiting, abdominal pain or tenderness, elevated transaminase level(s), or jaundice, and at least organism(s) seen on Gram stain and/or identified from intraabdominal fluid or tissue obtained during invasive procedure or from an aseptically-placed drain in the intraabdominal space, or organism(s) identified from blood by a culture or non-culture based microbiologic testing method, performed for purposes of clinical diagnosis or treatment and imaging test evidence suggestive of infection [2].

**SUPPLEMENTARY REFERENCES**

1. Singer M, Deutschman CS, Seymour CW, et al. The Third International Consensus Definitions for Sepsis and Septic Shock (Sepsis-3). JAMA. **2016**; 315(8):801–810.

2. CDC/NHSN Surveillance Definitions for Specific Types of Infections. **2021**; :30.

**Supp Figure 1.** A) Kaplan-Meier curve for 28-day intubation. B) Kaplan-Meier curve for 28-day ICU stay.


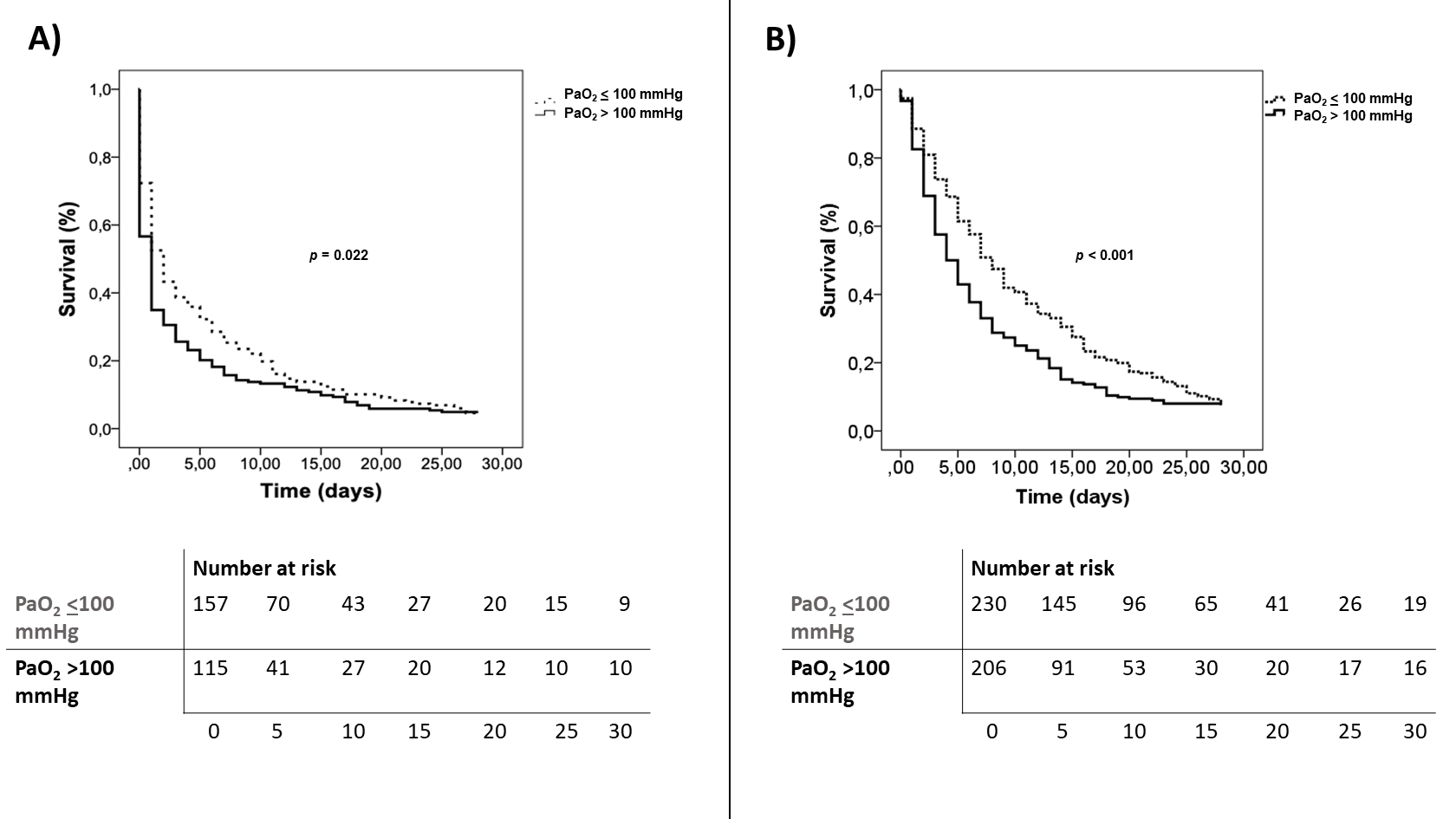


**Supp Figure 2.** Kaplan-Meier survival curves for 90-day mortality of the multivariate regression model.


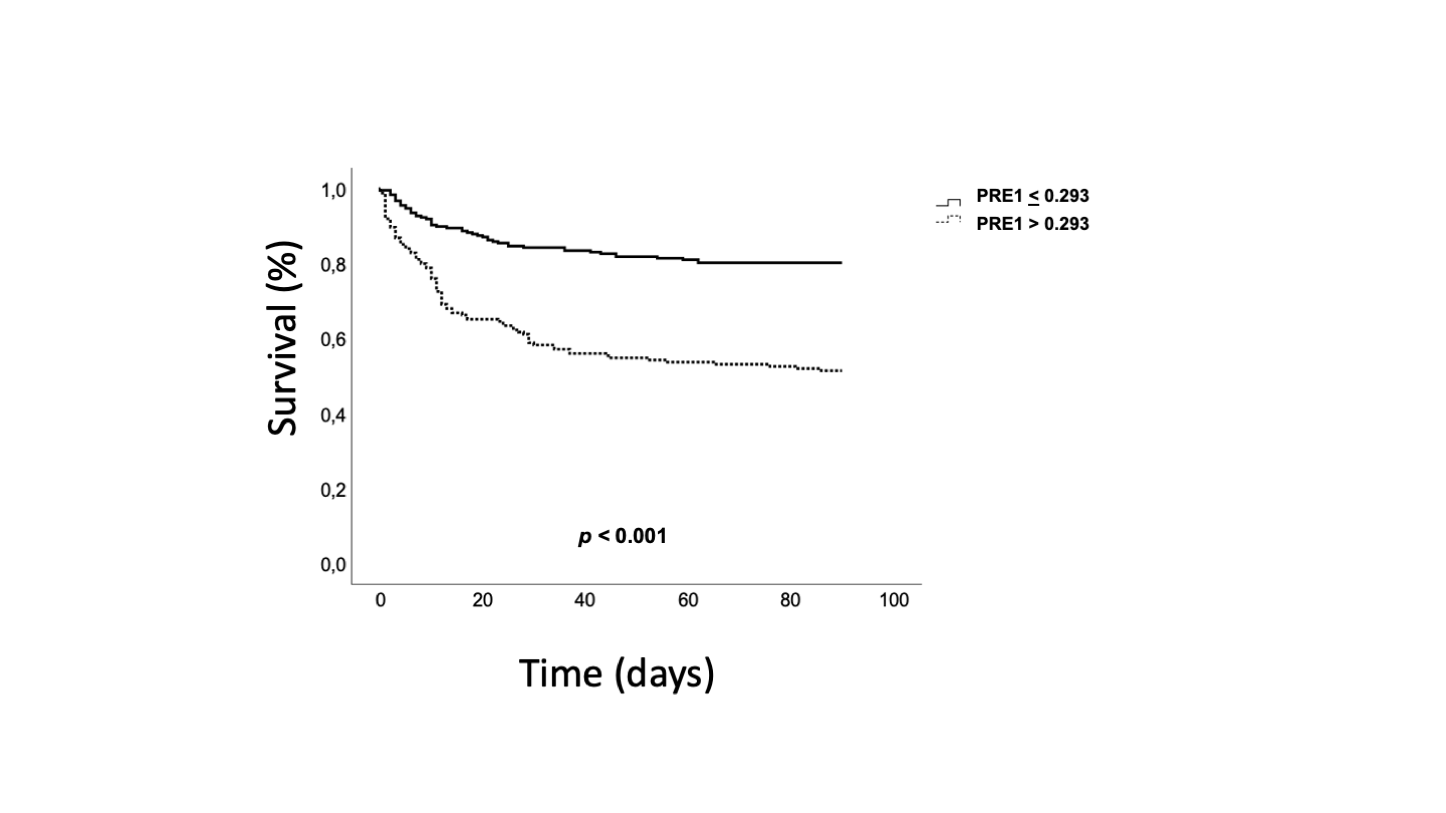


**Supp Table 1.** Univariate and multivariate analysis for evaluating the risk of mortality at 90 days excluding patients with severe hypoxemia (PaO_2_<60 mmHg) at the time of inclusion into the study.

|  | **Univariate analysis** | | | | **Multivariate analysis** | | | |
| --- | --- | --- | --- | --- | --- | --- | --- | --- |
|  | **OR** | **[CI 95%]** | | ***p*** | **OR** | **[CI 95%]** | | ***p*** |
| **Age** | 1.04 | 1.01 | 1.06 | 0.001 | 1.03 | 1.01 | 1.05 | 0.015 |
| **Chronic renal failure** | 3.55 | 1.80 | 6.98 | < 0.001 | 3.36 | 1.62 | 6.98 | 0.001 |
| **PCT (ng/mL) Ln** | 1.21 | 1.07 | 1.38 | 0.003 | 1.18 | 1.02 | 1.36 | 0.025 |
| **APACHE II> 19** | 3.49 | 2.03 | 6.02 | < 0.001 | 2.63 | 1.46 | 4.73 | 0.001 |
| **PaO_2_ > 100 mmHg** | 0.55 | 0.36 | 0.83 | 0.005 | 0.54 | 0.34 | 0.86 | 0.009 |

**Supp Figure 3.** Kaplan-Meier survival curves for 90-day mortality excluding patients with severe hypoxemia (PaO_2_<60 mmHg) at the time of inclusion into the study.


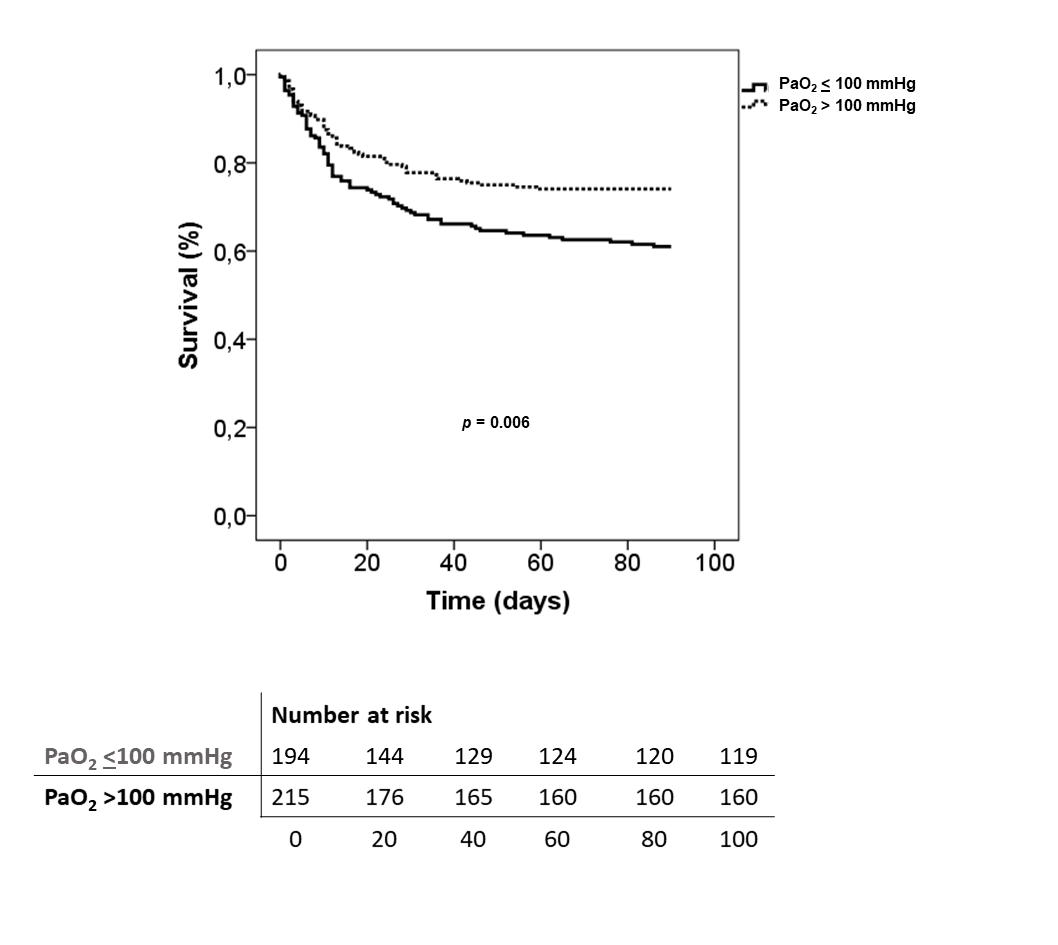


**Supp Figure 4.** A) Kaplan-Meier curve for 28-day intubation excluding patients with severe hypoxemia (PaO_2_<60 mmHg) at the time of inclusion into the study. B) Kaplan-Meier curve for 28-day ICU stay excluding patients with severe hypoxemia (PaO_2_<60 mmHg) at the time of inclusion into the study.

**
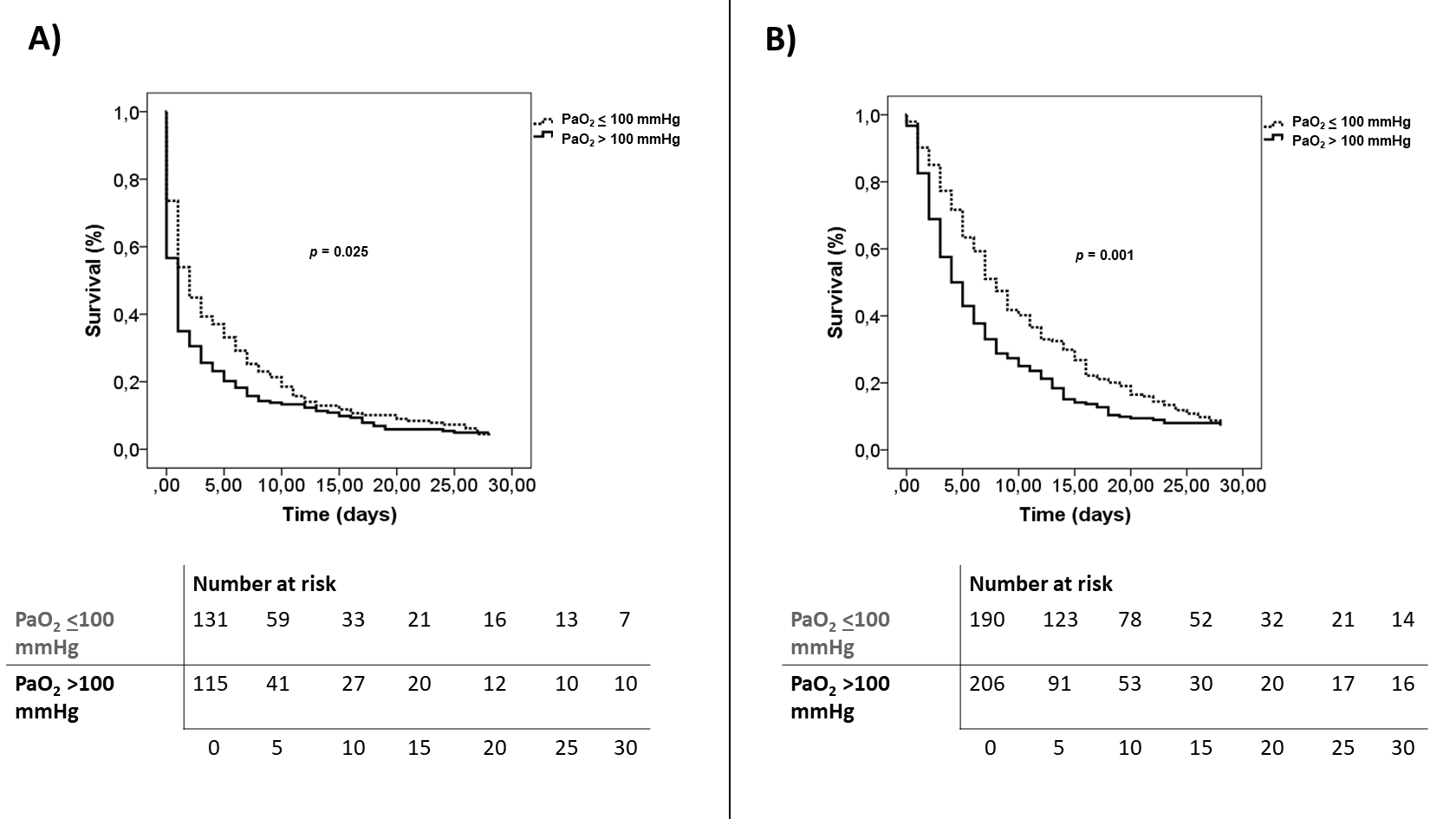
**

**Supp Figure 5.** Neutrophils’ bactericidal activity mediated by oxidative killing.


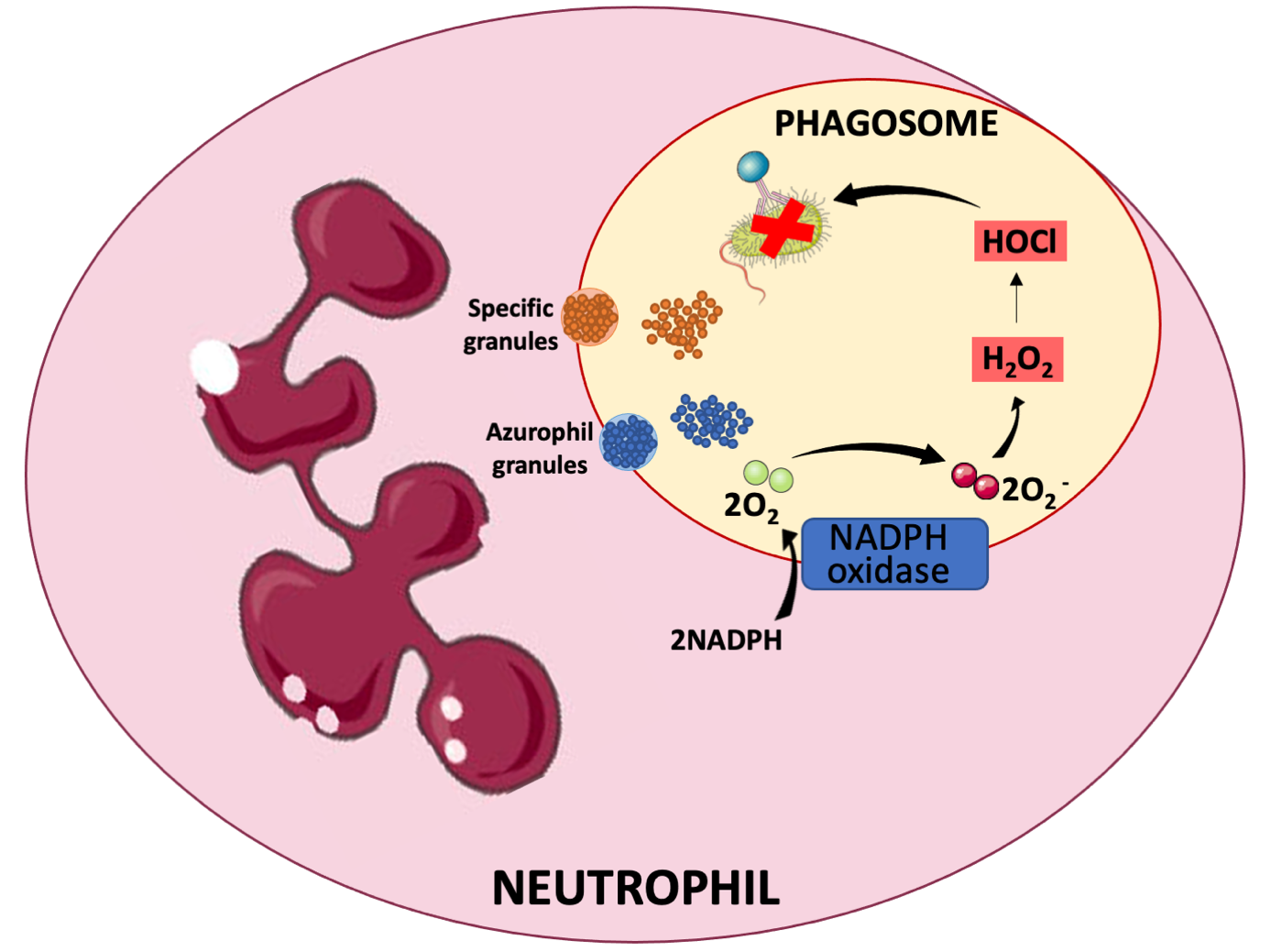

Supplement: Supplementary file 1 — Additional file 1: File 1. Supplementary methods regarding treatment, diagnosis and definitions of patients recruited for the study. Figure S1. (A) Kaplan–Meier curve for 28-day intubation. (B) Kaplan–Meier curve for 28-day ICU stay. Figure S2. Kaplan–Meier survival curves for 90-day mortality of the multivariate regression model. Table S1. Univariate and multivariate analysis for evaluating the risk of mortality at 90 days excluding patients with severe hypoxemia (PaO2 < 60 mmHg) at the time of inclusion into the study. Figure S3. Kaplan–Meier survival curves for 90-day mortality excluding patients with severe hypoxemia (PaO2 < 60 mmHg) at the time of inclusion into the study. Figure S4. (A) Kaplan–Meier curve for 28-day intubation excluding patients with severe hypoxemia (PaO2 < 60 mmHg) at the time of inclusion into the study. (B) Kaplan–Meier curve for 28-day ICU stay excluding patients with severe hypoxemia (PaO2 < 60 mmHg) at the time of inclusion into the study. Figure S5. Neutrophils’ bactericidal activity mediated by oxidative killing. [file 13054_2021_3875_MOESM1_ESM.docx]
